# Implication of IRF4 Aberrant Gene Expression in the Acute Leukemias of Childhood

**DOI:** 10.1371/journal.pone.0072326

**Published:** 2013-08-15

**Authors:** Maria Adamaki, George I. Lambrou, Anastasia Athanasiadou, Marianna Tzanoudaki, Spiros Vlahopoulos, Maria Moschovi

**Affiliations:** 1 Pediatric Hematology/Oncology Unit, First Department of Pediatrics, University of Athens, “Aghia Sofia” Children's Hospital, Athens, Greece; 2 Department of Immunology and Histocompatibility “Aghia Sofia” Children's Hospital, Athens, Greece; Queen's University Belfast, United Kingdom

## Abstract

The most frequent targets of genetic alterations in human leukemias are transcription factor genes with essential functions in normal blood cell development. The Interferon Regulatory Factor 4 (*IRF4*) gene encodes a transcription factor important for key developmental stages of hematopoiesis, with known oncogenic implications in multiple myeloma, adult leukemias and lymphomas. Very few studies have reported an association of *IRF4* with childhood malignancy, whereas high transcript levels have been observed in the more mature immunophenotype of ALL. Our aim was to investigate the expression levels of *IRF4* in the diagnostic samples of pediatric leukemias and compare them to those of healthy controls, in order to determine aberrant gene expression and whether it extends to leukemic subtypes other than the relatively mature ALL subpopulation. Quantitative real-time RT-PCR methodology was used to investigate *IRF4* expression in 58 children with acute leukemias, 4 leukemic cell lines and 20 healthy children. We show that aberrant *IRF4* gene expression is implicated in a variety of leukemic subtypes; higher transcript levels appear in the more immature B-common ALL subtype and in T-cell than in B-cell leukemias, with the highest expression levels appearing in the AML group. Interestingly, we show that childhood leukemia, irrespective of subtype or cell maturation stage, is characterised by a minimum of approximately twice the amount of *IRF4* gene expression encountered in healthy children. A statistically significant correlation also appeared to exist between high *IRF4* expression and relapse. Our results show that ectopic expression of *IRF4* follows the reverse expression pattern of what is encountered in normal B-cell development and that there might be a dose-dependency of childhood leukemia for aberrantly expressed *IRF4*, a characteristic that could be explored therapeutically. It is also suggested that high *IRF4* expression might be used as an additional prognostic marker of relapse at diagnosis.

## Introduction

Acute leukemia (AL) is regarded the most common type of malignancy in children. Despite the good overall response of childhood patients to current chemotherapeutic treatments and the long-term event-free survival rates exceeding 80% [1], [2], a significant percentage of the population show resistance to therapy and/or relapse, often with devastating consequences. This has highlighted the importance of devising new therapies that will target the resistant clones and increase overall survival. Of significant interest are the genetic alterations that might affect the characteristics of hematopoietic stem cells (HSCs). For example, it has already been demonstrated that a subset of genes expressed in normal HSCs are highly reactivated in leukemic stem cells (LSCs), thus indicating that the LSCs express the self-renewal-associated programme normally characterising the HSCs [3]. Such findings suggest that leukemic transformation of progenitor cells is associated with ectopic reactivation of the genes responsible for self-renewal. Indeed, the presence of *MLL* rearrangements in both acute lymphoblastic leukemia (ALL) and acute myeloid leukemia (AML) seems to relate with high level expression of genes normally expressed in HSCs during the early stages of hematopoiesis, such as *FLT3* and *HOXA9* [4]–[10]. Consequently, the identification of specific genetic aberrations that characterise leukemic cells, such as abnormal gene expression, can lead to a better understanding of what constitutes their unlimited self-renewing properties and subsequently reveal potential molecular targets for successful therapy.

The most frequent targets of genetic alterations in human leukemias are transcription factor genes with essential functions in normal blood cell development. The Interferon Regulatory Factor 4 (*IRF4*) gene encodes a transcription factor important for hematopoietic development and immune responses. It is essentially involved in all developmental stages within the B-cell lineage (except during the germinal center (GC) reaction), with known critical functions in at least 3 key developmental processes: the termination of the GC B-cell transcriptional program, immunoglobulin class-switch recombination, and plasma cell development [11]–[13]. *IRF4* is induced by mitogenic stimuli, such as antigen receptor engagement, lipopolysaccharides, and CD40 signaling [14], [15], which activate the Nuclear Factor-kappa B (NF-kB) pathway, ultimately leading to *IRF4* promoter activation [16]–[18]. Despite functioning as a tumor suppressor gene in early B-cell development [11], [19], *IRF4* is a well-established oncogene in multiple myeloma (MM) [20], with oncogenic implications extending to certain adult lymphomas and leukemias [13], [21]–[23]. High expression levels have been detected in chronic lymphocytic leukemia (CLL), where they have been linked to an unfavourable prognosis [24]–[27]. Very few studies have reported an association of *IRF4* with childhood malignancy. In childhood ALL, *IRF4* has been found significantly up-regulated in the more mature CD34-CD19^+^ LSC population [28], whereas others have reported that an intronic polymorphism of the gene relates to a greater risk association of males to the disease [29].

Considering the importance of *IRF4* in key developmental stages of hematopoiesis, as well as its oncogenic implication in malignancies such as lymphomas and chronic leukemias, investigating its expression patterns in childhood leukemias could help in the understanding of how deregulation can contribute to leukemogenesis. In this study we investigate the transcript levels of *IRF4* in the diagnostic samples of a cohort of 58 pediatric acute leukemia patients and 4 cell lines, including both ALL and AML, and compare them to those of healthy controls. Our aim is to determine whether aberrant *IRF4* expression extends to childhood AML or to leukemic subtypes other than the more mature CD34-CD19^+^ ALL population. In addition, we examine whether there are any correlations between gene expression and specific cytogenetic characteristics, as well as other diagnostic variables, such as age, gender, and karyotype. Finally, we investigate the prognostic impact of high *IRF4* expression in the clinical course of childhood leukemia, through possible correlations with minimal residual disease (MRD) and survival outcome.

## Materials and Methods

### Patient and control samples

Fifty eight (58) children were diagnosed with AL (52 with ALL and 6 with AML) at the Hematology/Oncology Unit of Athens University, at “Aghia Sofia” Children's Hospital, Athens, Greece. Bone marrow (BM) samples were collected at diagnosis and all patients were classified morphologically and immunologically. Patient data were as follows: males: 37, females: 21, median age at diagnosis (years): 4.72±4.32, median male age (years): 4.77±4.48, median female age (years): 4.07±3.89, median ALL age: 4.39±4.03, median AML age: 10.48±5.36. Patient clinical data are summarized in **Table S1**. BM samples were also collected from 20 healthy children to be used as negative controls for the qRT-PCR studies. Four childhood leukemia cell lines were also included in the study: REH (pre-B ALL), CCRF-CEM (T-cell ALL), CCRF-SB (T-cell ALL), and THP-1 (acute monocytic leukemia). All cell lines were obtained from the European Collection of Cell Cultures (ECACC, UK).

### Sample handling

Red Blood Cell Lysis Buffer (RBC Lysis Buffer) was added to every BM sample (2∶1 ratio), in preparation for RNA extraction. Every sample was vortexed and left at room temperature for 3–5 minutes to ensure sufficient lysis of red blood cells, then centrifuged at 3,000 rpm for 7 minutes, so that the white blood cells, intact, collected at the bottom of the tube, while the lysed red blood cells remained in the liquid supernatant. For immunophenotyping, samples were collected in EDTA tubes containing 1 ml of Hank's Balanced Salt Solution for every 1 ml of BM specimen. Specimens were analyzed within 3 hours post collection.

### RNA Extraction

Total RNA was extracted from BM leukocytes/myelocytes, using TRIzol reagent (Invitrogen, Inc.) according to the manufacturer's protocol. Extraction of RNA from the 4 cell lines was performed with the same protocol, only without the addition of RBC Lysis Buffer, for obvious reasons. RNA was treated with amplification grade DNase I to eliminate any residual genomic DNA from the sample and further purified with the RNeasy® Micro Kit (Qiagen Inc). RNA quantification measurements were performed on the NanoDrop® ND-1000 Spectrophotometer (Nanogen Inc.).

### Flow cytometry and Immunophenotype

Blast cell immunophenotyping was performed by direct immunofluoresence and 5-colour flow cytometry, on a FC500 instrument (Beckman Coulter, Brea CA). Apoptosis and necrosis measurements were performed as described previously [30].

BM blast cells were tested with a panel of monoclonal antibodies (MoAbs) (Beckman Coulter) against B- and T-lymphoid and myeloid-associated antigens to complement the immunological classification. Surface expression of CD45 (clone J33), CD34 (clone 581), HLADR (clones 9–49 (I3) and IMMU357), CD19 (clone J4.119), CD10 (clone ALB1), CD20 (clone Η299), CD7 (clone 3A1E-12H7), CD2 (clone SFCI3Pt2H9), CD5 (clone BL1a), CD1a (clone BL6), CD33 (clone D3HL60.251) and CD13 (clone SJ1D1) was determined in all specimens. Cytoplasmic μ chain expression was determined when indicated by a more mature B-cell phenotype, such as CD20 positivity. The T-ALL panel included additional CD4 (clone SFCI12T4D11), CD8 (clone SFCI21Thy2D3), cCD3, sCD3 (for both clone UCHT1) and TdT (pool HT-1, HT-3, HT-4) expression determination. The AML panel included, among others, the supplementary use of antibodies to cMPO (clone CLB-MPO 1), CD117 (clone 104D2D1), CD11b (clone Bear1), CD15 (clone 80H5), CD16 (clone 3G8), CD14 (cloneRMO52) and CD64 (clone 22). Additional indices, such as CD79α or CD22, were studied where necessary, as for example in cases of ambiguous diagnosis.

### Real Time Quantitative RT-PCR

Gene expression was investigated with the Real-Time Reverse Transcription PCR (qRT-PCR), using the Plexor™ One-Step qRT-PCR System kit (Promega Inc.) and the LightCycler 2 Instrument (Roche Diagnostics GmbH). Both the reverse transcriptase and DNA polymerase were added during the initial reaction setup, and the thermal cycler was programmed to perform the reverse transcription step, immediately followed by the thermal cycling program.

For each 25 µl PCR reaction, a 12.5 µl aliquot from the Plexor® Master Mix (2×) was added to a LightCycler® glass capillary (Roche Diagnostics GmbH), followed by 0.5 µl of RNasin® Plus RNase Inhibitor, 0.2 µl of ImProm-II™ Reverse Transcriptase, 2 µl of primers (1 µl Forward primer and 1 µl Reverse primer), 1 µl of RNA (20 ng/µl) and 8.8 µl of nuclease-free water. For the housekeeping gene reaction only 1 µl of primer was added, as both the Reverse and Forward primers were provided in the same tube by the manufacturer (Eurogentec S.A.).

The thermal cycling programme was as follows: 1 cycle of 45°C for 5 minutes (reverse transcription), 1 cycle of 95°C for 2 minutes (initial denaturation and inactivation of the reverse transcriptase), 40 cycles of 95°C for 5 seconds (denaturation) and of 63°C for 35 seconds (annealing and extension), and 60°C to 95°C, ramp 0.4°C/second intervals (melt temperature curve). The specificity of the PCR products was verified by gel analysis and products were shown to consist of only a single band.

The sequences of the primers in the 5′ to 3′ direction were as follows: *IRF4F*: 5′-AG-CGC-ATT-TCA-GTA-AAT-GTA-AAC-ACA-T-3′ and *IRF4R*: 5′-TCT-TGT-GTT-CTG-TAG-ACT-GCC-ATC-A-3′. Housekeeping genes *GAPDH* and *b-actin* were used for normalization purposes. In their majority experiments were performed with the *GAPDH* housekeeping gene.

Initially, method sensitivity was tested by creating a standard curve with kanamycin control RNA as provided by the manufacturer. The standard curve included six points at a final RNA concentration ranging from 50 ng to 0.0005 ng (ie. 0.5 pg) in 10-fold increments, therefore it was decided that for the patient and control samples a total of 20 ng would be sufficient for each reaction.

During the first 6 experiments (6 patients) reactions were performed in duplicates and experiments were performed in triplicates to assess intra- and inter-assay variability (no significant variability found). In all other experiments the patient and control samples were tested in triplicate reactions and experiments were performed once. The average value for all three reactions was used for quantification. Non-template negative control reactions were included with each run. All PCR products were electrophoresed on a 2% agarose gel to confirm successful amplification of the desired products.

### Real-Time Data pre-processing and Analysis

Real-time data were collected and pre-processed using the LightCycler Software Version 3.5 (Roche Diagnostics GmbH). Following this, data were exported to the Plexor™ Analysis Software (www.promega.com/plexorresources) (Promega, Inc.) and gene expression was obtained in the form of Ct values; this refers to the PCR cycle number during exponential amplification at which the product (measured in real-time by fluorescence emission) crosses an arbitrary threshold. Data pre-processing was performed with Microsoft® Excel.)

### Statistical Analysis

First of all an independent t-test was performed between patients and controls to assess whether there were any differences in the mean *GAPDH* expression levels (Ct values) between the two groups. The t-test failed to reveal a statistically reliable difference (p = 0.068), so we accepted all *GAPDH* Ct values as reliable normalization constants. A second t-test was then performed to compare the mean *IRF4* expression levels between the two groups, and a statistically significant difference was revealed (p = 0.003). The Ct value of *IRF4* (for every patient and healthy control) was then normalized against the Ct value obtained for the housekeeping gene, *GAPDH* (ie. ΔCt  =  Ct of *IRF4* – Ct of *GAPDH*) and the corrected gene expression levels were defined as the expression level for each gene divided by the *GAPDH* expression level. Taking the corrected expression levels (ΔCt) into account, each patient was tested for significance in differential expression using a z-test. Patients were regarded as significantly differentially expressing the gene if their z-score had a corresponding p-value of ≤0.05. T-tests were performed using the IBM SPSS Statistics program (IBM Corp. Released 2010. IBM SPSS Statistics for Windows, Version 19.0. Armonk, NY: IBM Corp.), whereas all other analyses were performed with Microsoft® Excel. Raw Data are included in **Table S2** (Raw Data for *IRF4*) and all statistical analyses performed on SPSS are included in **File S3** (Independent t-Tests for *IRF4*).

## Results

### Immunophenotypic and cytogenetic subtypes

Immunophenotypes, karyotypes and cytogenetics are also presented in **Table S1**. Our results are in agreement with other studies regarding the frequencies of the immunophenotypic groups [31], [32]. Similarly, our calculated cytogenetic frequencies also seem to be in agreement with those of other western countries [33].

### Quantitative Real Time RT-PCR analysis

**Figure 1:**
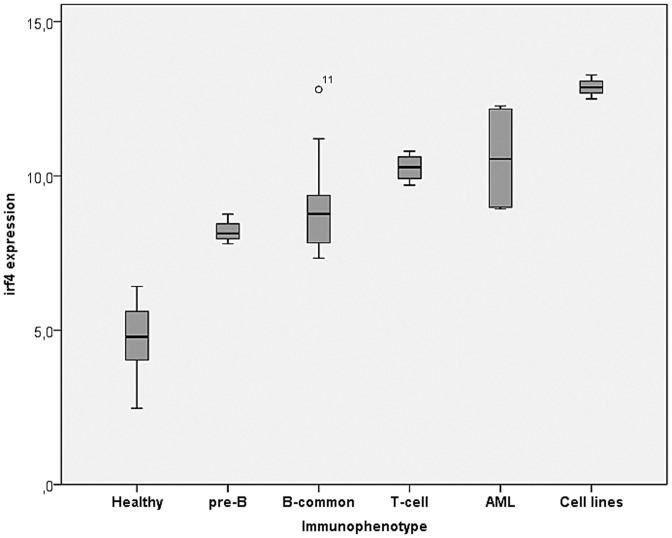
*IRF4* expression levels in various immunophenotypic groups.

A quantitative real-time RT-PCR assay was established for the gene of interest. *IRF4* was found to be differentially over-expressed, as compared to the healthy controls, in 30 patients (51.7% of total cohort): 26 ALL (18 B-common, 3 pre-B, 4 T-cell and 1 infant leukemia of unknown immunophenotype), 4 AML (1 with M1 and 3 with M5 maturation), and 3 of the 4 cell-lines: REH (pre-B ALL), CCRF-CEM (T-cell ALL) and THP-1 (AML) (**Figure 1**). The patients over-expressing *IRF4* are also highlighted in bold in **Table S1**. Five (5) of these 30 patients and the 3 cell lines mentioned above revealed a much higher expression ratio (R>11.6) than the rest (p = 0.000). In addition, the gene was found to be differentially down-regulated in 6 patients (∼10%) (**Table 1**). Therefore, patients were divided into 4 groups: subnormal ratio (ratio <2.4), normal (healthy) ratio (2.4≤ ratio <7), high ratio (7< ratio <11.6) and very high ratio (11.6< ratio). In the subnormal ratio group, the mean *IRF4* expression was significantly lower (p = 0.000) than in the healthy controls group (**File S3**). Overall, the gene was found up-regulated in a broader spectrum of leukemic subtypes and maturation stages than previously thought, with the highest transcript levels appearing in the AML group (**Figure 1**).

**Table 1:** *IRF4* expression levels in leukemia patients and cell lines.

	Subnormal ratio R<2.4	Normal ratio 2.4≤R<7	High ratio 7<R<11.6	Very high ratio 11.6<R	p-value*
Healthy donors (n = 20)	–	4.72±1.17 (n = 20)	–	–	
All patients & cell lines (n = 62)	0.89±0.73 (n = 6)	4.99±1.2 (n = 23)	**8.90**±**1.11 (n = 27)**	**12.6**±**0.44 (n = 6)**	**0.000**
Patients & cell lines with ALL (n = 55)	0.89±0.73 (n = 6)	5.02±1.2 (n = 21)	**8.89**±**1.16 (n = 25)**	**12.72**±**0.20 (n = 3)**	**0.000**
Pro-pre-B ALL (patients) (n = 2)	0.55 (n = 1)	4.80 (n = 1)	–	–	
B-common ALL (patients) (n = 35)	0.81±0.62 (n = 3)	4.69±1.28 (n = 14)	**8.77**±**1.11 (n = 17)**	**12.8 (n = 1)**	**0.000**
Pre-B ALL (patients) (n = 9)	1.17±1.27 (n = 2)	5.64±0.99 (n = 4)	**8.23**±**0.49 (n = 3)**	–	**0.000**
Pre-B ALL (cell lines) (n = 1)	–	–	–	**12.50 (n = 1)**	**0.000**
T-cell ALL (patients) (n = 5)	–	6.90 (n = 1)	**10.27**±**0.47 (n = 4)**	–	**0.000**
T-cell ALL (cell lines) (n = 2)	–	5.40 (n = 1)	–	**12.87 (n = 1)**	**0.000**
Unknown (N/A) ALL Patient (n = 1)	–	–	**7.35 (n = 1)**	–	**0.041**
Patients & cell lines with AML (n = 7)	–	4.77±0.85 (n = 2)	**8.98**±**0.07 (n = 2)**	**12.54**±**0.64 (n = 3)**	**0.001**
Patients with AML (n = 6)	–	4.77±0.85 (n = 2)	**8.98**±**0.07 (n = 2)**	**12.17**±**0.14 (n = 2)**	**0.000**
Cell lines with AML (n = 1)	–	–	–	**13.27 (n = 1)**	**0.000**

*the p-value has been calculated by taking into account both groups over-expressing IRF4 (i.e. both high and very high ratio), as compared to the healthy controls ratio.

### Correlation Analysis Between Gene Expression and Cytogenetic Abnormalities

From our group of pediatric patients found to over-express *IRF4*, only 8 (26.7%) appeared to have one of the commonly found cytogenetic translocations, and that was either *TEL-AML1* or *MLL* rearrangement, with the exception of one patient who was *BCR-ABL*+ve (**Table S1**). The majority of patients with high transcript *IRF4* levels (n = 22) did not have a detectable cytogenetic translocation at diagnosis or had additional cytogenetic abnormalities, such as extra gene copy numbers, deletions, etc. No apparent correlation was found between high gene transcript levels and specific cytogenetic abnormalities.

### Correlation Analysis Between Gene Expression and other diagnostic variables

No apparent correlation was found between abnormal *IRF4* expression and age, gender, or karyotype (data not shown).

### Correlation Analysis Between Gene Expression and MRD levels

No apparent correlation was found between abnormal *IRF4* expression and resistance to therapy (as measured in MRD level on day 30). Only 3 patients had detectable MRD on day 30 since initiation of treatment (NYII protocol for ALL and AML-BFM 2004 protocol for AML), one of whom relapsed, along with another patient who relapsed but who had no detectable MRD on day 30 (**Table S2**).

### Correlation Analysis Between Gene Expression and Overall Survival

**Figure 2:**
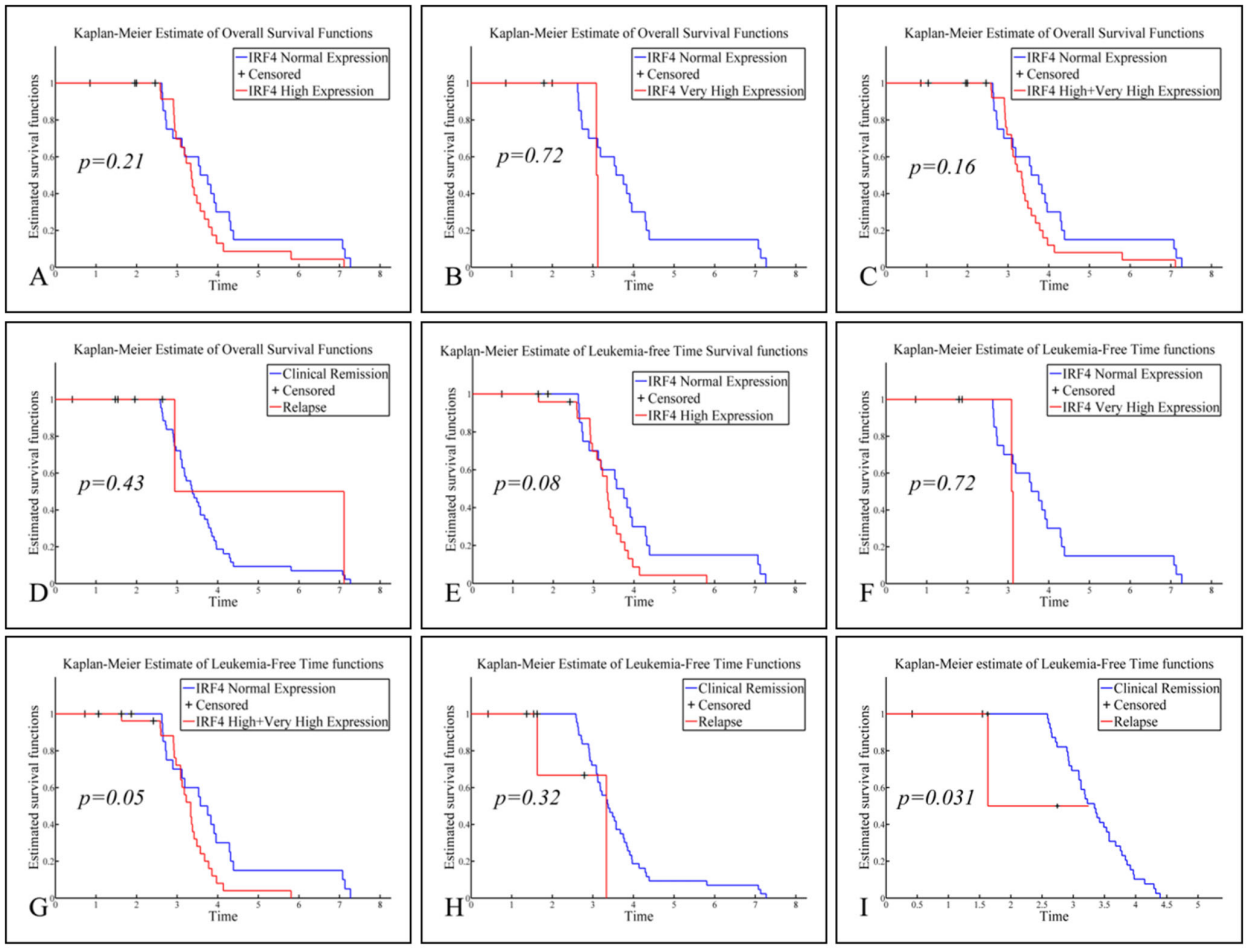
Overall survival (OS) and leukemia-free time analysis with respect to *IRF4* expression. Comparisons of OS included: normal levels of *IRF4* vs. high levels of *IRF4* (**A**), normal levels of *IRF4* vs. very high levels of *IRF4* (**B**), normal levels of *IRF4* vs. both high and very high levels of *IRF4* (**C**) and clinical remission vs. relapse irrespectively of *IRF4* expression (**D**). Similarly, the same analysis was performed for leukemia-free time and comparisons included: normal levels of *IRF4* vs. high levels of *IRF4* (**E**), normal levels of *IRF4* vs. very high levels of *IRF4* (**F**), normal levels of *IRF4* vs. both high and very high levels of *IRF4* (**G**) and clinical remission vs. relapse irrespectively of *IRF4* expression (**H**). Interestingly, when leukemia-free time was estimated within a certain time frame (3-year) high *IRF4* expression appeared to correlate with leukemia-free survival (**I**).

No significant correlation was found between abnormal *IRF4* expression and overall survival (OS) (as measured in years of survival from the diagnosis) on comparing patients with normal and patients with high *IRF4* expression (**Figure 2** **, A**–**H**). Please note that Patients 56 and 57 were diagnosed at a much earlier date (2003) and so were regarded as outliers and were not included in the analysis. Even though we have observed twice as many deaths (4 versus 2) in the patient group over-expressing *IRF4* (**Table S2**), the Kaplan Meier curves have not produced a statistically significant difference (p>0.05). The results for estimated leukemia-free time (i.e. time to relapse) were marginally significant, as it can be seen in **Figure 2** (**E & G**) (p = 0.08, CI = 90% and p = 0.05, CI = 95% respectively). Interestingly, when leukemia-free time was estimated within a specific time frame of 3-years (i.e. only the patients diagnosed in the years 2008, 2009 and 2010 were included in the analysis), a significant correlation appeared to exist between high *IRF4* expression and relapse (p = 0.03) (**Figure 2** **, I**).

## Discussion

In the present study we have found high *IRF4* transcript levels in a significant number of patients (51.7%) and in 3 of the 4 cell lines, as compared to the healthy controls. This up-regulation seems to associate with a variety of leukemic subgroups, possibly reflecting the many roles of *IRF4* in the immune system [12]. Considering that *IRF4* is essential for several stages of normal B- and T-cell activation and differentiation, it is only logical to assume that aberrations in the *IRF4* transcriptome would also reflect in lymphoid-cell associated malignancy.

A significant difference in *IRF4* transcript levels was observed between the pre-B and B-common cohorts, as well as between the total B- and T-cell ALL groups (**Figure 1**), with higher transcript levels appearing in the B-common and T-cell subtypes, respectively. In the past, however, *IRF4* mRNA expression has been found low in adult samples of T-cell ALL [34]. At this point it is difficult to validate the reasons for this difference, besides possible age-related dissimilarities in the biology of ALL, yet it points out that aberrant *IRF4* transcriptional activity is developmental-stage-specific as well as cell-type-specific. It has already been demonstrated that *IRF4* is expressed at varying levels throughout B-cell development, with expression peaking in plasma cells, therefore varying transcript levels in human malignancies could mirror *IRF4* expression in normal lymphoid activation and differentiation [35], [36]. However, in contrast to the Gene Expression Omnibus (GEO) data profiles from healthy tissues [37] and a recent study from le Viseur *et al* [28], in our leukemia samples we have observed that the greater the distance from a differentiated B-cell, the higher the level of *IRF4* expression. In other words, significantly higher transcript levels were observed in the B-common subgroup, than in the more mature pre-B subtype (CD34^dim/^-CD19^+^). This suggests that, at least for the particular patient cohort studied here, ectopic expression of *IRF4* does not exactly mirror the expression patterns encountered in normal B-cell development, but rather the situation seems to reverse. This observation could reflect differences in our experimental set-up, as opposed to the study from le Viseur *et al* [28], as we have not measured *IRF4* expression in flow-sorted subpopulations from the same diagnostic sample but have rather based our analyses on the overall prevailing immunophenotype for every patient. On the other hand, the 3 cell lines, despite being phenotypically distant from differentiated cells, appeared to express even higher levels of the gene than the patients (all 3 fell into the very high ratio >11.6); this possibly implies that in childhood leukemia, *IRF4* gene expression is not subject to the feedback regulation mechanisms that operate in normal tissues.

In the past, *IRF4* expression was regarded an almost exclusive characteristic of the lymphoid lineage [15], but recent data have also demonstrated an involvement in myeloid cell differentiation [38]–[40]. The implication is still not entirely understood, but ectopic expression of *IRF4* in myeloid progenitor cells in *vitro* was recently shown to inhibit myeloid cell growth and to tune the balance of lineage selection in myeloid progenitor cells by stimulating macrophage differentiation, whilst inhibiting neutrophil differentiation [41]. Subsequently, expression has been found down-regulated in chronic myeloid leukemias (CML), as well as in adult AML patient samples [34], [42], [43]. Here we report an association of *IRF4* up-regulation with childhood AML, as it is in this patient cohort that we observe the highest transcript levels, including the AML cell line. Interestingly, 2 of the 4 AML patients over-expressing *IRF4* fell into the very high ratio group (11.6< ratio) (**Table 1**) and were in fact positive for M5 maturation, whereas high expression was also observed in the M1 stage (without maturation). This could also highlight the role of *IRF4* in early myeloid cell differentiation and, subsequently, in myeloid leukemia without maturation but, since the number of observed cases here is small, no such conclusions can be made at this point. Analysis of a larger cohort of patients and of myeloid cell populations at various maturation stages should be able to confirm or reject the validity of this notion. The opposing expression levels between adult and childhood AML (low versus high) are most probably the result of the different pathogenetic mechanism that discriminates between adult and childhood leukemias.

Significant differentially low *IRF4* expression, as compared to the healthy controls (p = 0.000) was observed in 6 of the patient samples that we analysed (∼10% of total patients), all of which were B-cell ALL positive. This observation is consistent with the presence of a B-lineage-restricted repressor on the promoter of the *IRF4* gene, which can become over-expressed in a cross-section of B-lineage leukemia [44], [45]. Since *IRF4* binds to its own promoter region, creating the potential for positive auto-regulation, its expression can reach levels that activate the repressor [17]. Other leukemic subtypes seem to lack the repressor and therefore do not exhibit abnormally low *IRF4* expression. Furthermore, at least one micro-RNA species has been identified as an *IRF4* repressor. In the B-cell lineage, hsa-miR-125b (GC-enriched hsa-miR-125b) has been shown to directly down-regulate the expression of *IRF4* and of the repressor PRDM1/BLIMP1 [46]. While *IRF4* expression in non-B-cell leukemia can be deregulated toward higher levels, in B-lineage leukemia activation of the repressor may also facilitate a reduction in *IRF4* transcript levels below the expected average.

Overall, as it can be seen in **Figure 1**, it seems that childhood leukemia, irrespective of subtype or cell maturation stage, is characterised by a minimum of approximately twice the amount of *IRF4* gene expression encountered in healthy children (as measured in corrected expression levels (ΔCt)). This might suggest that there is a dose-dependency of childhood leukemia for ectopically expressed *IRF4*, a characteristic that could be explored further for its therapeutic potential. A therapy based on *IRF4*-knockout would most likely be hindered by the potentially detrimental side effects. *IRF4*-deficient mice show profound defects of the immune system, such as impaired lymphocyte function, inability to generate cytotoxic, antibody and antitumor responses, accompanied by a severe reduction in serum immunoglobulin concentrations [14]. Surprisingly, mice lacking one allele of *IRF4* appear phenotypically normal, whereas a 50% knockdown of *IRF4* mRNA and protein has proved effective in clearing MM cell lines [14], [47]. Perhaps the same should be applied to leukemic cells in order to evaluate whether modified or partial silencing of the gene appears to have an inhibitory or apoptotic effect, and thus whether it opens up a new opportunity of a therapy aimed at *IRF4*.

No significant correlation was found between high *IRF4* expression and OS (**Figure 2** **, A**–**H**). This could be due to the small number of patients included in the analysis or perhaps to the fact that for most of these patients OS could not be calculated in terms of a 3-year or a 5-year survival. Whereas a marginally significant correlation appeared to exist between abnormal *IRF4* expression and estimated leukemia-free time (p = 0.08 and p = 0.05) (**Figure 2** **, E & G**), a significant correlation appeared (p = 0.03) when leukemia-free time was estimated within a specific time frame of 3 years (**Figure 2** **, I**). This implies that high *IRF4* expression might be used as an additional prognostic marker of relapse at the diagnosis of childhood acute leukemias. Further analyses on a larger number of patients over-expressing *IRF4*, over a longer period of time (i.e. 5-year survival), should be able to confirm this observation in the near future.

Additional efforts should be made in order to identify the putative targets of *IRF4* and to determine the ones directly regulated by it. A number of proteins have already been identified as repressors in B-cells and found to interact with *IRF4*: PU.1, Spi-B, E47, NFAT, Stat6, Bcl-6 and Blimp-1 [14], [38], [48]–[50]. In addition, CD40 signaling has been found to directly induce the expression of *IRF4* in GC B-cells which, in turn, binds to the promoter region of the *BCL-6* gene to repress its expression [51]. We are currently investigating the expression patterns of other hematopoietic progenitor genes in a larger cohort of pediatric patients in an attempt to gain a better understanding of how deregulated transcription on key signaling pathways affects the malignant phenotype and disease progression in childhood leukemia.

## Conclusions

In summary, our study shows that aberrant *IRF4* gene expression is implicated in a variety of leukemic subtypes, possibly reflecting the many roles of the gene in the immune system. In contrast to other studies, we observe higher transcript levels in patients characterised by a more immature immunophenotype, and in T-cell leukemias than in leukemias of the B-lineage, implying that in childhood leukemia, *IRF4* gene expression is not subject to the feedback regulation mechanisms that operate in normal tissues. In addition, ectopic expression of *IRF4* does not seem to be a specific feature of lymphoid leukemia, but it also extends to myeloid leukemia, as it was in our AML group that the highest expression levels were observed. Again, our results are in contrast to other studies documenting a down-regulated *IRF4* expression in adult myeloid leukemias [34], [42], [43], even though this is most probably due to age-related dissimilarities and a different pathogenetic leukemia mechanism. Interestingly, we show that childhood leukemia, irrespective of subtype or cell maturation stage, is characterised by a minimum of approximately twice the amount of *IRF4* gene expression encountered in healthy children. This implies that there might be a dose-dependency of the disease for aberrantly expressed *IRF4*, a characteristic that could be explored therapeutically. In addition, a statistically significant correlation was found between high *IRF4* expression and relapse, suggesting a prognostic value of *IRF4* in the clinical course of childhood leukemias. Data from the past few years indicate that many important regulators of HSC development are also implicated in the abnormal self-renewal capacity of the LSCs. Only through the identification of these regulators, and of their downstream targets, will we gain a better understanding of what constitutes their unlimited self-renewing properties and subsequently reveal potential molecular targets for successful therapy.

## Supporting Information

File S1 **Independent t-Tests for patients and cell lines over-expressing** ***IRF4*** **(both high and very high expression) and for patients under-expressing** ***IRF4*** **.**
(DOC)

Table S1 **Clinical data.**
(DOC)

Table S2 **Raw data for** ***IRF4*** **.**
(XLSX)
