# Supplementary material for: Implication of IRF4 Aberrant Gene Expression in the Acute Leukemias of Childhood
Source: PLoS One. 2013 Aug 15;8(8):e72326. doi: 10.1371/journal.pone.0072326 (PMC3744475; doi:10.1371/journal.pone.0072326)
Supplement: File S1 — Independent t-Tests for patients and cell lines over-expressing IRF4 (both high and very high expression) and for patients under-expressing IRF4 . (DOC) [file pone.0072326.s001.doc]

File S3:

Independent t-Tests for patients and cell lines over-expressing IRF4 (both high and very high expression) and for patients under-expressing IRF4


All patients and cell lines

Group Statistics	
	Group	N	Mean	Std. Deviation	Std. Error Mean	
IRF4_expression_DeltaCT	Controls	20	4.7215	1.17210	.26209	
	All patients & cell lines	33	9.5758	1.78239	.31028	


Independent Samples Test	
	Levene's Test for Equality of Variances	t-test for Equality of Means	
	F	Sig.	t	df	Sig. (2-tailed)	Mean Difference	Std. Error Difference	95% Confidence Interval of the Difference	
								Lower	Upper	
IRF4_expression_DeltaCT	Equal variances assumed	4.524	.038	-10.823	51	.000	-4.85426	.44852	-5.75471	-3.95381	
	Equal variances not assumed			-11.952	50.584	.000	-4.85426	.40615	-5.66981	-4.03870	


Patients and cell lines with ALL


Group Statistics	
	Group	N	Mean	Std. Deviation	Std. Error Mean	
IRF4_expression_DeltaCT	Controls	20	4.7215	1.17210	.26209	
	Patients & cell lines with ALL	28	9.3011	1.62966	.30798	


Independent Samples Test	
	Levene's Test for Equality of Variances	t-test for Equality of Means	
	F	Sig.	t	df	Sig. (2-tailed)	Mean Difference	Std. Error Difference	95% Confidence Interval of the Difference	
								Lower	Upper	
IRF4_expression_DeltaCT	Equal variances assumed	2.062	.158	-10.727	46	.000	-4.57957	.42691	-5.43890	-3.72024	
	Equal variances not assumed			-11.324	45.991	.000	-4.57957	.40440	-5.39360	-3.76555	


B-common ALL


Group Statistics	
	Group	N	Mean	Std. Deviation	Std. Error Mean	
IRF4_expression_DeltaCT	Controls	20	4.7215	1.17210	.26209	
	B-common ALL	18	8.9972	1.43473	.33817	


Independent Samples Test	
	Levene's Test for Equality of Variances	t-test for Equality of Means	
	F	Sig.	t	df	Sig. (2-tailed)	Mean Difference	Std. Error Difference	95% Confidence Interval of the Difference	
								Lower	Upper	
IRF4_expression_DeltaCT	Equal variances assumed	.179	.675	-10.102	36	.000	-4.27572	.42325	-5.13411	-3.41733	
	Equal variances not assumed			-9.994	32.927	.000	-4.27572	.42784	-5.14625	-3.40520	


Pre-B ALL

(same as on high ratio)

Group Statistics	
	Group	N	Mean	Std. Deviation	Std. Error Mean	
IRF4_expression_DeltaCT	Controls	20	4.7215	1.17210	.26209	
	pre-B ALL	3	8.2333	.49319	.28474	

Independent Samples Test	
	Levene's Test for Equality of Variances	t-test for Equality of Means	
	F	Sig.	t	df	Sig. (2-tailed)	Mean Difference	Std. Error Difference	95% Confidence Interval of the Difference	
								Lower	Upper	
IRF4_expression_DeltaCT	Equal variances assumed	1.991	.173	-5.041	21	.000	-3.51183	.69668	-4.96065	-2.06302	
	Equal variances not assumed			-9.075	6.345	.000	-3.51183	.38700	-4.44644	-2.57722	


T-cell ALL

(same as on high ratio)


Group Statistics	
	Group	N	Mean	Std. Deviation	Std. Error Mean	
IRF4_expression_DeltaCT	Controls	20	4.7215	1.17210	.26209	
	T-cell ALL	4	10.2650	.46580	.23290	


Independent Samples Test	
	Levene's Test for Equality of Variances	t-test for Equality of Means	
	F	Sig.	t	df	Sig. (2-tailed)	Mean Difference	Std. Error Difference	95% Confidence Interval of the Difference	
								Lower	Upper	
IRF4_expression_DeltaCT	Equal variances assumed	2.720	.113	-9.178	22	.000	-5.54350	.60400	-6.79613	-4.29087	
	Equal variances not assumed			-15.811	12.296	.000	-5.54350	.35062	-6.30540	-4.78160	


Unknown Immunophenotype ALL (patient)

(same as on high ratio)


Group Statistics	
	Group	N	Mean	Std. Deviation	Std. Error Mean	
IRF4_expression_DeltaCT	Controls	20	4.7215	1.17210	.26209	
	Unknown Immunophenotype	1	7.3500	.	.	

Independent Samples Test	
	Levene's Test for Equality of Variances	t-test for Equality of Means	
	F	Sig.	t	df	Sig. (2-tailed)	Mean Difference	Std. Error Difference	95% Confidence Interval of the Difference	
								Lower	Upper	
IRF4_expression_DeltaCT	Equal variances assumed	.	.	-2.189	19	.041	-2.62850	1.20105	-5.14232	-.11468	
	Equal variances not assumed			.	.	.	-2.62850	.	.	.	


Pre-B ALL (cell line)

(same as on very high ratio)


Group Statistics	
	Group	N	Mean	Std. Deviation	Std. Error Mean	
IRF4_expression_DeltaCT	Controls	20	4.7215	1.17210	.26209	
	pre-B ALL	1	12.5000	.	.	


Independent Samples Test	
	Levene's Test for Equality of Variances	t-test for Equality of Means	
	F	Sig.	t	df	Sig. (2-tailed)	Mean Difference	Std. Error Difference	95% Confidence Interval of the Difference	
								Lower	Upper	
IRF4_expression_DeltaCT	Equal variances assumed	.	.	-6.476	19	.000	-7.77850	1.20105	-10.29232	-5.26468	
	Equal variances not assumed			.	.	.	-7.77850	.	.	.	


T-cell ALL (cell line)

(same as on very high ratio)


Group Statistics	
	Group	N	Mean	Std. Deviation	Std. Error Mean	
IRF4_expression_DeltaCT	Controls	20	4.7215	1.17210	.26209	
	T-cell ALL	1	12.8700	.	.	


Independent Samples Test	
	Levene's Test for Equality of Variances	t-test for Equality of Means	
	F	Sig.	t	df	Sig. (2-tailed)	Mean Difference	Std. Error Difference	95% Confidence Interval of the Difference	
								Lower	Upper	
IRF4_expression_DeltaCT	Equal variances assumed	.	.	-6.784	19	.000	-8.14850	1.20105	-10.66232	-5.63468	
	Equal variances not assumed			.	.	.	-8.14850	.	.	.	


Patients and cell lines with AML


Group Statistics	
	Group	N	Mean	Std. Deviation	Std. Error Mean	
IRF4_expression_DeltaCT	Controls	20	4.7215	1.17210	.26209	
	All patients & cell lines with AML	5	11.1140	2.00072	.89475	


Independent Samples Test	
	Levene's Test for Equality of Variances	t-test for Equality of Means	
	F	Sig.	t	df	Sig. (2-tailed)	Mean Difference	Std. Error Difference	95% Confidence Interval of the Difference	
								Lower	Upper	
IRF4_expression_DeltaCT	Equal variances assumed	5.417	.029	-9.448	23	.000	-6.39250	.67658	-7.79212	-4.99288	
	Equal variances not assumed			-6.856	4.709	.001	-6.39250	.93234	-8.83448	-3.95052	


Patients with AML


Group Statistics	
	Group	N	Mean	Std. Deviation	Std. Error Mean	
IRF4_expression_DeltaCT	Controls	20	4.7215	1.17210	.26209	
	Patients with AML	4	10.5750	1.84401	.92200	


Independent Samples Test	
	Levene's Test for Equality of Variances	t-test for Equality of Means	
	F	Sig.	t	df	Sig. (2-tailed)	Mean Difference	Std. Error Difference	95% Confidence Interval of the Difference	
								Lower	Upper	
IRF4_expression_DeltaCT	Equal variances assumed	3.666	.069	-8.319	22	.000	-5.85350	.70360	-7.31267	-4.39433	
	Equal variances not assumed			-6.107	3.501	.006	-5.85350	.95853	-8.67137	-3.03563	


Cell lines with AML

(same as on very high ratio)


Group Statistics	
	Group	N	Mean	Std. Deviation	Std. Error Mean	
IRF4_expression_DeltaCT	Controls	20	4.7215	1.17210	.26209	
	AML Cell Lines	1	13.2700	.	.	


Independent Samples Test	
	Levene's Test for Equality of Variances	t-test for Equality of Means	
	F	Sig.	t	df	Sig. (2-tailed)	Mean Difference	Std. Error Difference	95% Confidence Interval of the Difference	
								Lower	Upper	
IRF4_expression_DeltaCT	Equal variances assumed	.	.	-7.118	19	.000	-8.54850	1.20105	-11.06232	-6.03468	
	Equal variances not assumed			.	.	.	-8.54850	.	.	.	


COMPARISON between patients with HIGH and VERY HIGH ratio


Group Statistics	
	Group	N	Mean	Std. Deviation	Std. Error Mean	
IRF4_expression_DeltaCT	High ratio	27	8.8970	1.11454	.21449	
	Very high ratio	6	12.6300	.43731	.17853	


Independent Samples Test	
	Levene's Test for Equality of Variances	t-test for Equality of Means	
	F	Sig.	t	df	Sig. (2-tailed)	Mean Difference	Std. Error Difference	95% Confidence Interval of the Difference	
								Lower	Upper	
IRF4_expression_DeltaCT	Equal variances assumed	3.744	.062	-7.986	31	.000	-3.73296	.46745	-4.68634	-2.77959	
	Equal variances not assumed			-13.376	21.313	.000	-3.73296	.27907	-4.31281	-3.15312	


COMPARISON between patients with SUBNORMAL ratio and Controls


Group Statistics	
	Group	N	Mean	Std. Deviation	Std. Error Mean	
IRF4_expression_DeltaCT	Controls	20	4.7215	1.17210	.26209	
	Subnormal ratio	6	.8867	.73159	.29867	


Independent Samples Test	
	Levene's Test for Equality of Variances	t-test for Equality of Means	
	F	Sig.	t	df	Sig. (2-tailed)	Mean Difference	Std. Error Difference	95% Confidence Interval of the Difference	
								Lower	Upper	
IRF4_expression_DeltaCT	Equal variances assumed	1.413	.246	7.523	24	.000	3.83483	.50971	2.78284	4.88683	
	Equal variances not assumed			9.651	13.551	.000	3.83483	.39736	2.97992	4.68975	
