# Supplementary material for: Implication of IRF4 Aberrant Gene Expression in the Acute Leukemias of Childhood
Source: PLoS One. 2013 Aug 15;8(8):e72326. doi: 10.1371/journal.pone.0072326 (PMC3744475; doi:10.1371/journal.pone.0072326)
Supplement: Table S1 — Clinical data. (DOC) [file pone.0072326.s002.doc]

**Table S1. Clinical data**

| **Patient/  Cell-line**  **code** | **Gender** | **Diagnosis** | **Age at diagnosis (yrs)** | **blast count (%)** | **WBC (cells/ul)** | **Immunophe-notypic classification** | **Karyotype** | **Cytogenetics** | **Additional Cytogenetics** |
| --- | --- | --- | --- | --- | --- | --- | --- | --- | --- |
| **57** | **Μ** | **infant ALL** | **0,45** | **86,000** | **51300** | **N/A** | **N/A** | **N/A** |  |
| J1 | M | infant ALL | 1,00 | 92,683 | 88010 | pro-pre-B | 48,XY,+X,der(1),t(2;9)(p10;q10),  +6,t(11;19)(q23;p13.3)[14]  /46,XY[1] | MLL |  |
| T | F | infant ALL | 0,16 | 97,405 | 738000 | pro-pre-B | 46,XX,t(4;11)(q21;q23)[10] | MLL |  |
| **83** | **F** | **ALL** | **2,41** | **72,707** | **7330** | **B-common** | **56,XX,+X,+4,+4,+6,+10,+15,+17,+18,+21,+21[3]/46,XX[7]** |  | **3 copies AML1 (60.4%) &**  **4 copies of AML1 (9.9%)** |
| 55 | Μ | ALL | 13,95 | 88,000 | 82600 | B-common |  | BCR/ABL |  |
| 44 | M | ALL | 13,08 | 84,548 | 20920 | B-common | 46,XY[10] |  |  |
| **61** | **F** | **ALL** | **6,05** | **85,911** | **10670** | **B-common** | **46,XX,t(9;22)(q34;q11),i(9)(q10)[3]/ 46,XX[3]** | **BCR/ABL** |  |
| **69** | **F** | **ALL** | **4,10** | **82,970** | **1430** | **B-common** | **46,XX,i(7)(q10)[19]/46,XX[1]** |  | **Aneuploidy at chr 7 (95%)** |
| **78** | M | ALL | 7,77 | 66,971 | 14970 | B-common | 46,XY[20] |  |  |
| **111** | **M** | **ALL** | **4,68** | **79,826** | **9430** | **B-common** | **47,ΧΥ,+18[7]/46,ΧΥ[14]** |  | **Trisomy 18** |
| **O-57** | **M** | **ALL** | **7,33** | **42,000** | **46000** | **B-common** | **87,<4n>,XY,+Y,+1,+1,+2,+2,+3,+3,+4,+4,+5,+5,+6,+6,+7,+7,+8,+8,+9,+9,+10,+11,+11,+12,+12,+13,+14,+15,+16,+16,+17,+17,+18,+18,+19,+19,+20,+20,+21,+21,+22,+22[5]/46,XY[5]** | **TEL/AML1** |  |
| V2 | F | ALL | 3,21 | 65,045 | 22910 | B-common | 46,XX[20] | TEL/AML1 |  |
| 86 | M | ALL | 1,75 | 85,998 | 6460 | B-common | 47,XY,+21[8]/46,XY[12] |  | Trisomy 21 (40%),  4 copies of AML1 (0.3%) |
| 123 | M | ALL | 13,05 | 80,837 | 3690 | B-common | 46,XY[20] | TEL/AML1 |  |
| **145** | **F** | **ALL** | **2,23** | **67,200** | **14130** | **B-common** | **46,XY[5]** | **TEL/AML1** |  |
| **130** | **M** | **ALL** | **3,29** | **96,826** | **9580** | **B-common** | **46,XY[20]** |  |  |
| **147** | **M** | **ALL** | **2,92** | **81,700** | **9730** | **B-common** | **51,XY,+X,+6,+14,+17,+21[1]/ 46,XY[14]** |  | **positive for D835 mutation**  **on FLT3 (no ITDs on FLT3)** |
| **148** | **Μ** | **ALL** | **3,62** | **88,900** | **42560** | **B-common** | **52,XY,+4,+6,+10,+17,+21,+21[5] /46,XY[2]** |  | **5 copies AML1 (64.2%),**  **4 copies AML1 (23.13%), 6 copies AML1 (4.5%),**  **3 copies AML1 (1.5%)** |
| 155 | Μ | ALL | 2,19 | 96,800 | 4700 | B-common | 61,XY,+Y,+4,+6,+8,+10,+10,+13,+14,+15,+17,+18,+21,+21,+21,  +21[3] |  | 6 copies AML1 (98.3%) |
| 163 | Μ | ALL | 2,70 | 64,700 | 20570 | B-common | 61,XY,+X,+4,+5,+6,+?7,+8,+10,+12,+14,+16,+17,+18,+21,+21,+21[4]/46,XY[6] |  | 3 copies TEL, 4 copies AML1 (24.6%), 3 copies TEL, 5 copies AML1 (68.6%) |
| 170 | Μ | ALL | 1,34 | 87,313 | 17054 | B-common | 46,XY[10] | TEL/AML1 |  |
| **O-47** | **F** | **ALL** | **5,80** | **49,457** | **4370** | **B-common** | **56,XX,+X,dup(1)(q31q41),+4?,+6,+8,+10,+11,+14,+15,+21,**  **+21[1]/ 46,XX[19]** |  | **4 copies AML1 (19%),**  **3 copies MLL (13.7%)** |
| O-42 | F | ALL | 5,29 | 10,000 | 2380 | B-common | 55,XX;  Trisomy 4, 7, 8, 14, 15, 17, 21, X | TEL/AML1 | Trisomy chr 4, 7, 8, 14, 15, 17, 21, X |
| O-73 | Μ | ALL | 3,95 | 23,000 | 11500 | B-common | 46, XY | TEL/AML1 |  |
| **119** | **M** | **ALL** | **6,06** | **90,917** | **4100** | **B-common** | **55,XY,+X,+4,+6,+10,+14,+17,+18,+21,+21[1]/46,XY[15].**  **nucish(AMLx4) [116/122]** |  | **4 copies AML1 (95.1%),**  **1 copy TEL (37.4%)** |
| **143** | **M** | **ALL** | **4,76** | **95,665** | **108300** | **B-common** | **46,XY,add(19)(q13)[4]/46,XY[3]** | **TEL/AML1** |  |
| **105** | **F** | **ALL** | **3,30** | **7,889** | **30820** | **B-common** | **46,XX[4]** | **TEL/AML1** |  |
| **146** | **M** | **ALL** | **5,28** | **81,700** | **8050** | **B-common** | **46,XY[5]** |  | **3 copies PBX1 (48%), 4 copies AML1 (85%),**  **5 copies AML1 (8%)** |
| 74 | F | ALL | 13,72 | 84,018 | 5940 | B-common | 46,XX,i(7)(q10),der(19)t(1;19)(q23;p13)[1]/46,XX,der(4)t(1;4)(q21;p16), der(16)t(1;16)(q21;q24),  der(19)t(1;19) (q23;p13)[2]/ 46,XX[12] | E2A/PBX1 | 1 copy MLL (2.2%) |
| 98 | M | ALL | 14,21 | 90,649 | 21330 | B-common | 46,XY,i(9)(q10),del(13)(q12q22), der(19)t(1;19)(q23;p13)[6] /46,sl,del(13)(q12q22)[3]/ 46,XY[1] |  |  |
| **99** | **M** | **ALL** | **4,07** | **94,513** | **3570** | **B-common** | **46,XY[20]** |  |  |
| **109** | **F** | **ALL** | **6,02** | **90,893** | **6070** | **B-common** | **46,XX[15]** |  |  |
| **140** | **M** | **ALL** | **2,80** | **82,625** | **53740** | **B-common** | **46,XY[1]** |  | **4 copies AML1 (84%),**  **3 copies AML1 (5%)** |
| 165 | Μ | ALL | 1,09 | 70,397 | 79520 | B-common | 46,XY[10] | TEL/AML1 |  |
| **60** | **F** | **infant ALL** | **1,01** | **94,000** | **42370** | **B-common** | **46,XX,add(9)(p13),**  **del(9)(p13p24),**  **t(X;13)(q13;q22)[15]/46,XX[1]** |  | **Loss of both copies of gene**  **p16 at 9p21 (87.4%)** |
| O-48 | M | ALL | 14,96 | 78,945 | 92960 | B-common | 45,XY,t(9;22)(q34;q11.2),  der(9)t(9;13)(p12;q1)  del(11)(q13qter),13[3] /46,sl,+mar[6]/45,sl1,mar,add(22)  (p11.1)[3]/88,sl x 2,-17,-Y[3] | BCR/ABL |  |
| S | M | ALL | 7,47 | 83,279 | 55050 | pre-B | 46,XY,t(3;4)(q21;p16),  t(8;16;11)(q13;q22;p11.2)[6]  /46,XY[4] |  | 3-break rearrangement: t(3;4)(q21;p16), t(8;16;11)(q13;q22;p11.2) |
| **76** | **F** | **ALL** | **9,31** | **62,353** | **3000** | **pre-B** | **46,XX,t(1;7)(p32;q32),t(2;8)(p13;q22),add(12)(p13),20,+21[16]**  **/46,XX[4]** |  | **3 copies AML1 (78.7%)** |
| 68 | F | infant ALL | 0,70 | 89,273 | 74800 | pre-B | 46,XX[15] | MLL |  |
| **110** | **F** | **ALL** | **3,25** | **27,207** | **1220** | **pre-B** | **47,XX,+21c[6]** |  |  |
| 131 | M | ALL | 8,78 | 30,330 | 9560 | pre-B | 46,XY,der(19)  t(1;19)(q23;p13)[12]/  46,XY[13] | E2A/PBX1 | 3 copies ABL (5.3%) |
| 164 | Μ | ALL | 2,37 | 90,100 | 18320 | pre-B | 46, XY[7] | TEL/AML1 |  |
| 169 | F | ALL | 6,79 | 83,279 | 360 | pre-B | 53,XX,+18,+19,+20,+21,+22,+22,+mar[3]/46,XX[18] |  | 3 copies TEL & 3 copies  AML1 (8.4%) 3 copies TEL & 4 copies  AML1 (89.2%) 3 copies MLL (94%) |
| 172 | Μ | ALL | 7,42 | 27,432 | 12200 | pre-B | 61,XY,+X,+Y,+3,+4,+5,+6,+10,+11,+17,+17,+18,+18,+20,+21,+21[1]/46,XY[9] |  | 4 copies AML1 (17%),  3 copies MLL (14%),  4 copies PBX1 (8%) |
| **177** | **Μ** | **ALL** | **4,77** | **89,314** | **7170** | **pre-B** | **53,ΧΥ,+Χ,+6,+10,+18,+21**  **+dup(21)(q22),+mar[5]**  **/46,XY[10]** |  | **Extra copies AML1 (72.5%): 6-15 copies** |
| O-43 | F | ALL | 2,30 | 80,000 | 146000 | pre-B | 46, XX |  |  |
| O-63 | F | ALL | 4,07 | 84,200 | 98000 | pre-B | 46, XX |  |  |
| 70 | M | ALL | 13,23 | 85,041 | 128500 | cortical T | 46, XY |  | TCRA/D rearrangement:  t(14q11) (8.9%) |
| **108** | **M** | **ALL** | **2,96** | **84,385** | **143500** | **cortical T** | **46,XY,del(6)(q12q22),i(9)(q10q10),t(11;14)(p13;q11)[7]**  **/46,XY[8]** |  | **TCRA/D rearrangement (0.8%)  3 copies ABL (0.9%) Chromosome losses : part of chr 6, part of chr 9,  translocation between chr 11 & chr 17** |
| **134** | **M** | **ALL** | **11,84** | **90,000** | **547300** | **cortical T** | **46,XY,del(6)(q13q27),**  **der(13)t(6;13)(q13p11.2)[8]**  **/46,XY[1]** |  | **Loss of long arm chr 6 (q13-q27); derivative chr 13  (mutual translocation between chr 6 (q13) & chr 13 (p11.2))** |
| **91** | **F** | **ALL** | **5,83** | **82,734** | **164100** | **medullary T** | **46,XX,t(1;19)(p32;q13.113.3),**  **t(1;11)(q32;p13),**  **del(6)(q23q27)[7]/46,XX[8]** |  |  |
| **124** | **M** | **ALL** | **5,79** | **86,472** | **97810** | **medullary T** | **46,XY,del(7)(q11.2),add(10)(p13)[6]/46,XY[14]** |  |  |
| **CCRF-CEM** | **F** | **cell-line ALL** | **4,00** | **100,000** | **5000** | **T-cell** |  | **t(8;9)(p11;p24)** |  |
| CCRF-SB | M | cell-line ALL | 11,50 | 100,000 | 5000 | T-cell |  | N/A |  |
| **REH** | **F** | **cell-line ALL** | **N/A** | **100,000** | **5000** | **Non-B/Non-T** |  | **TEL/AML1** |  |
| 80 | M | AML | 9,51 | 22,410 | 3390 | M1 | 48,XY,add(3)(p25),+8,inv(16)(p13q22), +22[2]/47,sl,-8[11]  /47,sdl1,+8,-22[7] | inv16 | CBFB rearrangement (92.4%) 3 copies of chr 8 (22.6%) |
| **174** | **F** | **AML** | **3,86** | **90,000** | **7400** | **M1** | **46,ΧΧ[4]** |  |  |
| 101 | F | AML | 16,18 | 99,000 | 2490 | M2 | 45,X,-X,t(8;21)(q22;q22)[10] | AML1/ETO | 4 copies ABL (90.7%) |
| **56** | **Μ** | **AML** | **12,82** | **64,000** | **1300** | **M5** |  | **MLL** |  |
| **133** | **M** | **infant AML** | **0,41** | **95,000** | **20840** | **M5** | **46,XY,inv(11)(q14q23)[20]** | **MLL** |  |
| **154** | **Μ** | **AML** | **11,44** | **89,700** | **232300** | **M5** | **47,XY,+8,der(9)t(9;11)(p10;q10)**  **t(9;11)(p22;q23),der(11)t(11;17)**  **(q10;p10),der(17)t(9;17)**  **(p10;q10)t(9;11)(p22;q23) [15]** | **MLL** |  |
| **THP-1** | **M** | **cell-line AML** | **1,00** | **100,000** | **5000** | **M5** |  | **t(9;11)** |  |
